# Novel Splicing Mutation in* B3GAT3* Associated with Short Stature, GH Deficiency, Hypoglycaemia, Developmental Delay, and Multiple Congenital Anomalies

**DOI:** 10.1155/2017/3941483

**Published:** 2017-11-28

**Authors:** Samuel Bloor, Dinesh Giri, Mohammed Didi, Senthil Senniappan

**Affiliations:** ^1^School of Life Sciences, University of Liverpool, Liverpool, UK; ^2^Institute of Child Health, University of Liverpool, Liverpool, UK; ^3^Department of Endocrinology, Alder Hey Children's NHS Foundation Trust, Liverpool, UK

## Abstract

*B3GAT3*, encoding *β*-1,3-glucuronyltransferase 3, has an important role in proteoglycan biosynthesis. Homozygous* B3GAT3* mutations have been associated with short stature, skeletal deformities, and congenital heart defects. We describe for the first time a novel heterozygous splice site mutation in* B3GAT3* contributing to severe short stature, growth hormone (GH) deficiency, recurrent ketotic hypoglycaemia, facial dysmorphism, and congenital heart defects. A female infant, born at 34 weeks' gestation to nonconsanguineous Caucasian parents with a birth weight of 1.9 kg, was noted to have cloacal abnormality, ventricular septal defect, pulmonary stenosis, and congenital sensorineural deafness. At 4 years of age, she was diagnosed with GH deficiency due to her short stature (height < 2.5 SD). MRI of the pituitary gland revealed a small anterior pituitary. She has multiple dysmorphic features: anteverted nares, small upturned nose, hypertelorism, slight frontal bossing, short proximal bones, hypermobile joints, and downslanting palpebral fissures. Whole exome sequencing (WES) was performed on the genomic DNA from the patient and biological mother. A heterozygous mutation in* B3GAT3* (c.888+262T>G) in the invariant “GT” splice donor site was identified. This variant is considered to be pathogenic as it decreases the splicing efficiency in the mRNA.

## 1. Introduction

Proteoglycans are an influential component of the extracellular matrix, orchestrating the cell-cell and cell-matrix interactions [1]. Defects in the biochemical machinery, which produce proteoglycans, can lead to severe multisystem disorders. Cell signalling pathways, most notably developmental pathways, may become disrupted and processes such as skeletal development and cardiovascular maturation will halt before they are fully complete [2]. Proteoglycans are produced through the secretory pathway in the endoplasmic reticulum followed by the construction of the glycosaminoglycan (GAG) side chain within the Golgi complex. Multiple posttranslational modifications occur in the Golgi complex, including the addition of disaccharides as well as epimerisation and sulfation of saccharide units, all performed by various glycosyltransferases, epimerases, and sulfotransferases [3].

*β*-1,3-Glucuronyltransferase 3 (GlcAT-I), encoded by* B3GAT3*, is located on chromosome 11q12.3 [4] and consists of 335 amino acids with one N-linked glycan chain [5]. GlcAT-I is a glucuronyltransferase involved in the biosynthesis of GAG-protein linkers for proteoglycans. Specifically, GlcAT-I contributes to the addition of the terminal four saccharides, xylose-galactose-galactose-glucuronic acid, hence its presence in the* cis-*Golgi [6]. The addition of this tetrasaccharide provides an external face for binding by extracellular signals.

Homozygous mutations in* B3GAT3* have previously been reported throughout the literature in patients with Larsen-like syndrome. Larsen syndrome has previously been characterised in association with proteoglycan synthesis related mutations,* B4GALT7*, and consists of phenotypic characteristics such as short stature, prominent forehead, and dislocations at multiple joints (knees, hips, elbows, and fingers) [7].

In this report, we describe, for the first time, a novel heterozygous splice site mutation in* B3GAT3 *contributing to severe short stature, growth hormone (GH) deficiency, facial dysmorphisms, and congenital heart defects amongst other symptoms.

## 2. Case Presentation

A female child born at 34 weeks' gestation to nonconsanguineous Caucasian parents with unremarkable antenatal history was noted to have a posterior cloaca requiring reconstructive surgery at 2 years of age. She has multiple dysmorphic features such as anteverted nares, small upturned nose, hypertelorism, slight frontal bossing, short proximal bones (femur, humerus) hypermobile joints, and downslanting palpebral fissures. She also has congenital sensorineural deafness, ventricular septal defect, and pulmonary stenosis, which required surgical correction. The phenotypic features are summarised in Table 1.

At 4 years of age, she was diagnosed with growth hormone (GH) deficiency due to her severe short stature (<2.5 SDS) and subsequently she was commenced on GH treatment. An MRI of her pituitary gland showed a small anterior pituitary and the other pituitary hormones were within the normal range. Investigations into her recurrent hypoglycaemic episodes following a prolonged fasting for 19 hours were consistent with ketotic hypoglycaemia (Table 2).

There is a history of short stature and dysmorphism present on the paternal side. There was also a previous stillborn elder sibling with midline cleft palate, absent uvula, small jaw, depressed nasal bridge, abnormalities on MRI brain such as absence of the inferior cerebellar vermis, partial agenesis of corpus callosum, congenital heart defects, and short bones.

CGH microarray did not reveal any copy number variants. A targeted exome sequencing of 70 genes associated with disorders of ketogenesis, ketolysis, carbohydrate metabolism, fatty acid oxidation defects, and hyperammonaemia did not identify any pathogenic mutations.

## 3. Methods

Whole exome sequencing (WES) was performed on the genomic DNA of the patient and biological mother after obtaining written and informed consent. We were unable to obtain samples from the father. The study was given favourable ethical opinion by the North West-Liverpool Central Research Ethics Committee (REC Reference: 15/NW/0758) and the Clinical Research Business Unit at Alder Hey Children's NHS Foundation Trust, Liverpool, UK, granted site study approval. Informed and written consent were obtained from the parents. Genomic DNA was extracted from the child and her biological mother. Exons were captured using SureSelect XT Human All Exon V5 capture library and DNA sequencing was carried out using the Illumina HiSeq4000 at 2 × 150 bp paired-end sequencer. The sequence data were aligned to the reference genome (GRCh37/hg19). The variants present in at least 1% minor allele frequency in 1000 Genomes Project, dbSNP142, and NHLBI ESP exomes were excluded. The predicted deleterious variants included nonsynonymous coding, splice site, frameshift, and stop gain variants.

**Figure 1:**
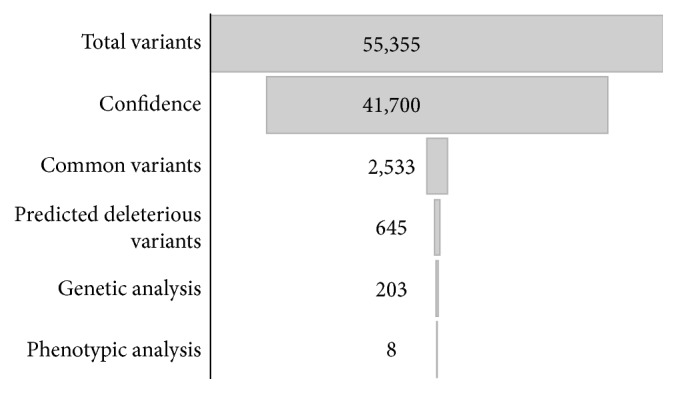
Ingenuity Variant Analysis (IVA) filtering schematic to determine genes of interest in the patient. Genes were filtered based upon confidence that the sequence was correctly sequenced, how common the gene is in the wider gene pool, a prediction of the variants' deleterious effect, and then the type of genetic mutation that it is. Genecards.org was then used to determine the phenotypic relevance of the gene to the symptoms of our patient.

Ingenuity Variant Analysis (IVA) bioinformatic software was used to develop a filtering system (Figure 1) in order to determine a small pool of genes, which are suspected to be pathogenic, causing the phenotypic features of the patient. Confidence values were set to a call quality of 20. Common variants included variants present in at least 1% minor allele frequency in the following databases: Allele Frequency Community, 1000 Genomes Project, ExAC, and the NHLBI ESP exomes. Followed by a prediction of the deleterious effect of the mutation using in silico tools such as PolyPhen, SIFT for SNPs, and MaxENT scan splice site mutations, the variants were categorised as either pathogenic or likely pathogenic. Finally, a genetic analysis was performed looking for homozygous, compound heterozygous, haploinsufficient, hemizygous, het-ambiguous, and heterozygous mutations. Subsequent research was performed upon the filtrate with genecards.org to determine any relevance to the symptoms of the patient.

## 4. Results

Of the 203 genetic variants that IVA filtered as likely to be pathogenic, genes such as* COL24A1, PLXND1, TECRL, EBF2, ABLIM1, PRDM10*, and* POSTN* were filtered out as they did not segregate with the patient phenotype following a detailed review of the biological information available from the current literature [8–14].

A heterozygous mutation in* B3GAT3* (c.888+262T>G) in the invariant “GT” splice donor site was identified. In silico modelling of this variant categorised the variant as pathogenic. This variant decreases the splicing efficiency of the mRNA as predicted by a MaxEntScan score decrease of 100% (from 11.01 to −0.14). MaxEntScan is an in silico splicing defect prediction tool used to analyse the affinity of an intronic sequence to the splicing machinery [15]. A decrease of 100% suggests that the splice site is completely lost, thus incurring a frameshift resulting in the formation of truncated protein.

## 5. Discussion

*B3GAT3* transcribes the 335-amino-acid glucuronyltransferase I (GlcAT-I) protein which catalyses the final step in proteoglycan biosynthesis through the addition of a xylose-galactose-galactose-glucuronic acid tetrasaccharide linkage molecule [16]. Homozygous missense mutations in* B3GAT3 *have previously been described as “linkeropathies.” Glycosaminoglycan linkeropathies are characterised by their enzymatic inability to synthesise the common linker region, which joins the core protein with its respective glycosaminoglycan side chain [17]. Proteoglycans are crucial for effective communication between cells. Disruption of the linkage region caused by mutations in* B3GAT3* has been reported to cause severe developmental defects.

**Figure 2:**
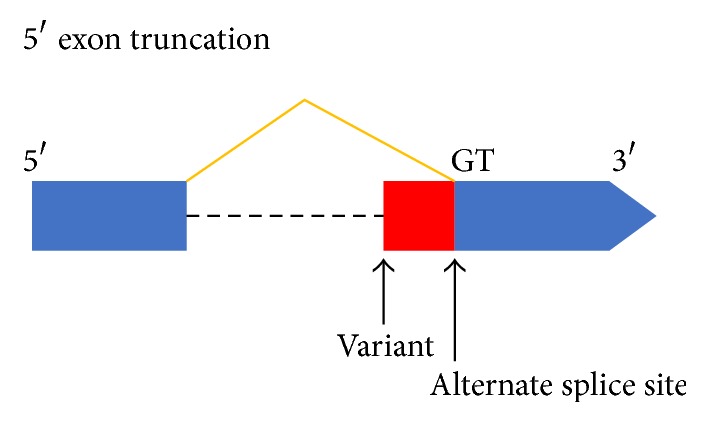
Schematic representation of the exon-intron structure in B3GAT3 depicting the position of the splice site variant and the GT splice donor site. The variant results in the creation of an alternative splice site.

We describe, for the first time, a novel heterozygous splice site mutation in* B3GAT3* (c.888+262T>G) in the invariant “GT” splice donor site (Figure 2). We hypothesise that the resultant truncated protein as a result of the splice site mutation leads to incomplete biosynthesis of the xylose-galactose-galactose-glucuronic acid terminus of the glycosaminoglycan side chain of the proteoglycan, due to a possible dominant negative effect interfering with dimerization and the resultant decrease in the enzyme activity.

The phenotype of our patient aligns with several other phenotypic features described to be associated with* B3GAT3* mutation (Table 1). All cases of* B3GAT3* have short stature, anteverted nares, downslanting palpebral fissures, and ventricular septal defects. However, these have all been associated with homozygous missense mutations such as c.671 T>A (p.Leu224Gln) [2], c.830 G>A (p.Arg277Gln) [1], and c. 667 G>A (p.Gly223Ser) [18]. For the first time, we describe a patient with splice site mutation in* B3GAT3* that is contributing to the phenotype. Our patient also has GH deficiency, showing a good response to treatment. The association between GH deficiency and* B3GAT3* mutation is currently unclear. However the association of developmental syndromes with GH/IGF1 (Insulin Growth Factor-1) abnormalities is expanding. One recent example is the association between Bainbridge Ropers syndrome and primary IGF1 deficiency [19].

In addition to some similarities in phenotypic features shown in Table 1, our patient also has growth hormone deficiency and recurrent ketotic hypoglycaemia. An initial targeted exome sequencing experiment was conducted to discover any mutated genes involved in ketogenesis, ketolysis, carbohydrate metabolism, fatty acid oxidation defects, and hyperammonaemia but no pathogenic mutations were identified. This is also the first time that GH deficiency is being reported in association with* B3GAT3 *mutation.

It is noteworthy to mention that the patient's biological father was also short with facial dysmorphism and short bones. Unfortunately, it was not possible to obtain DNA sample from the father. Besides, a history of stillborn elder sibling with facial dysmorphism, short bones, and heart defects suggests a likely strong penetrance of a monogenic genetic aetiology in the family. We acknowledge that genetic analysis in the biological father and the elder sibling would have convincingly established the underlying monogenic aetiology. However, we were limited due to the nonavailability of the samples from the father and the stillborn elder sibling.

## 6. Conclusion

*B3GAT3*, encoding *β*-1,3-glucuronyltransferase 3, has an important role in proteoglycan biosynthesis. Homozygous* B3GAT3* mutations have been associated with short stature, skeletal deformities, and congenital heart defects. A heterozygous* B3GAT3* mutation (c.888+262T>G) in the invariant “GT” splice donor site was identified in this study which is considered to be pathogenic as it decreases the splicing efficiency in the mRNA. Further functional studies might be useful to fully characterise the role of this splice site variant mutation in* B3GAT3*.

## Figures and Tables

**Table 1:** Comparison of known B3GAT3 phenotypes (+: expresses phenotype; −: does not express phenotype).

Phenotype	Our Patient	Yauy et al. (2017)		Baasanjav et al. (2011)		Job et al. (2016)	von Oettingen et al. (2014)	Jones et al. (2015)
Number of patients	1	6		5		1	1	1
*Skeletal malformations*								
Short stature	+	−	(0/6)	+	(5/5)	−	+	+
Fractures	−	+	(4/6)			+	−	+
Anteverted nares	+			+	(4/5)	−	+	+
Small upturned nose	+					+	+	+
Hypertelorism	+							+
Frontal bossing	+						+	
Short proximal bones	+					+	+	
Hypermobile joints	+							
Dislocating joints	−	+	(3/6)	+		+	+	
Joint laxity	−					+	+	
Diffuse demineralisation	−			+		+	−	+
Downslanting palpebral fissures	+			+	(3/5)	+		
*Congenital heart defects*		+	(3/7)					
Ventricular septal defect	+			+	(2/5)		−	+
Pulmonary stenosis	+						−	
Bicuspid aortic valve	−			+	(3/5)	+	−	
Aortic root dilation	−			+	(3/5)		+	
Mitral valve prolapse	−			+	(4/5)		−	
*Neurological*								
Small anterior pituitary	+			−		−	−	
Partially empty sella							+	
Other features								
TSH abnormality	−					+		
Cognitive delay	−					−	+	
Stillborn sibling	+							
GH deficiency	+							
Congenital sensorineural deafness	+							+
Ketotic hypoglycaemia	+							

**Table 2:** Results of investigations following a 19-hour fast.

Lab blood glucose	2.3 mmol/L
Insulin	<14 pmol/L
C-peptide	<33 pmol/L
Plasma free fatty acids	2673 umol/L
3-Hydroxybutyrate	1205 umol/L
Plasma free carnitine	13.2 umol/L
17 OHP	<1 nmol/L
Plasma amino acids	Normal
Urinary organic acids	Normal
